# Tissue Engineered Scaffolds for an Effective Healing and Regeneration: Reviewing Orthotopic Studies

**DOI:** 10.1155/2014/398069

**Published:** 2014-08-27

**Authors:** Silvia Baiguera, Luca Urbani, Costantino Del Gaudio

**Affiliations:** ^1^INAIL-DIPIA, Via Alessandria 220E, 00198 Rome, Italy; ^2^Surgery Unit, Institute of Child Health and Great Ormond Street Hospital, University College London, 30 Guilford Street, London WC1N 1EH, UK; ^3^University of Rome “Tor Vergata”, Department of Enterprise Engineering, Intrauniversitary Consortium for Material Science and Technology (INSTM), Research Unit “Tor Vergata”, Via del Politecnico 1, 00133 Rome, Italy

## Abstract

It is commonly stated that tissue engineering is the most promising approach to treat or replace failing tissues/organs. For this aim, a specific strategy should be planned including proper selection of biomaterials, fabrication techniques, cell lines, and signaling cues. A great effort has been pursued to develop suitable scaffolds for the restoration of a variety of tissues and a huge number of protocols ranging from* in vitro* to* in vivo* studies, the latter further differentiating into several procedures depending on the type of implantation (i.e., subcutaneous or orthotopic) and the model adopted (i.e., animal or human), have been developed. All together, the published reports demonstrate that the proposed tissue engineering approaches spread toward multiple directions. The critical review of this scenario might suggest, at the same time, that a limited number of studies gave a real improvement to the field, especially referring to* in vivo* investigations. In this regard, the present paper aims to review the results of* in vivo* tissue engineering experimentations, focusing on the role of the scaffold and its specificity with respect to the tissue to be regenerated, in order to verify whether an extracellular matrix-like device, as usually stated, could promote an expected positive outcome.

## 1. Introduction

**Figure 1:**
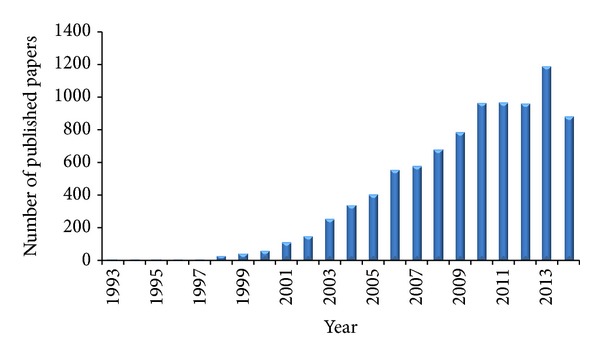
Timeline of published papers (number per year) from the PubMed website using the key “scaffold tissue engineering” (updated to July, 2014).

A simple search on the PubMed website using the key “scaffold tissue engineering” gave 8948 results (July, 2014), the first two papers being published in 1993, characterized by a linear-like distribution starting from the year 2000 (Figure 1). This occurrence suggests that, after an initial pioneering period, tissue engineering has been rapidly developed as an emerging research field with relevant implications for the enhancement of clinical treatments and the improvement of quality of life of a patient. Clearly, this can be considered a rough and not targeted bibliographic research that can be surely refined in order to highlight subtle and specific aspects related to this innovative and promising multidisciplinary approach, but it can give an idea, at the same time, of the great effort in the field. Now, after 20 years, a critical consideration about the overall findings collected so far can support the questions “where are we now?” and “have we enough information to move toward this or that direction?”. We hope to have a preliminary answer at the end of this review, since a definitive one would be unreasonable. For this aim, the present review focuses on the role of the scaffold in tissue engineering to be considered not just as a passive support for cell seeding but as an active platform that can effectively contribute to tissue regeneration and host integration. It is usually stated that such a scaffold should mimic the natural ECM of the tissue to be healed, but what this exactly means is the objective of this paper and, therefore, we will try to furnish a critical review of the literature data. One of the great expectations from tissue engineering is the potential to develop functional organs to overcome the current limitations related to the shortage of donor organs, incompatibility problems, and the detrimental effects of long-term use of immunosuppressive drugs after transplantation [1, 2]. The technical approach to address this issue is summarized into the well-known tissue engineering paradigm [3], and one of its key points specifically focuses on the definition of a viable and instructive scaffold for cell seeding, proliferation, migration, and differentiation in the case of stem cells. This statement implies a careful selection of: (i) the material(s) for scaffold fabrication, either synthetic or naturally derived, (ii) the most suitable technique that allows to deal with a substrate morphologically and mechanically similar to the ECM to be replaced, thus avoiding any mismatch with the surrounding tissue, (iii) possible surface treatments that can confer a positive biochemical profile to elicit a significant biological response, and (iv) drugs and/or growth factors to be loaded into the scaffold and subsequently released to enhance the final performance, avoiding, for instance, any side effect that can limit the therapeutic efficacy, for example, prevention of platelet adhesion on the luminal surface of tissue engineered vascular grafts [4].

Reasonably, due to the wide range to be covered and in order to furnish a clear scenario of the potential of a proper scaffold for tissue engineering applications, the main inclusion (or exclusion) criteria that led to the paper selection, here critically presented, were strictly related to the* in vivo* studies, preferentially focusing on orthotopic implantations. The rationale for this choice is expected to underline the findings that have already demonstrated promising results for the clinical translation of the tissue engineering approach. To provide a comprehensive survey on the topic, this review will firstly introduce a section on the common features of the ECM. Thereafter, currently investigated tissue engineered approaches, reporting only* in vivo* experimentations on tissue/organ and focusing on the properties of the scaffolds involved in the healing process, will be presented. Finally, a general discussion will resume the results with the aim to identify, if possible, the most promising strategies that can prompt an effective clinical translation.

## 2. The Extracellular Matrix: A Model for Tissue Engineered Scaffolds?

The ECM is the basic microstructure of each tissue and organ. It is usually referred to as one of the pivotal elements when a reliable tissue engineering strategy is needed. This relies on the fact that the replication of its specificity represents a key factor for the development of a scaffold that could provide a suitable microenvironment for a functional physioanatomic district to substitute the failing one.

The ECM functions as a structural as well as a signaling scaffold for cells, influencing cell behavior in terms of differentiation, proliferation, survival, and migration. It is a heterogeneous composition of proteoglycans, proteins, and signaling molecules. Structural proteins, such as collagen, elastin, and reticular fibers, are organized in an interwoven network of fibers and fibrils and provide architectural rigidity and mechanical support [5]. This fibrous architecture has also an important role in mechanotransduction: it deforms viscoelastically to external and internal stresses allowing cells to respond to mechanical stresses [6]. The ECM nonfibrous components, mostly glycosaminoglycans, regulate turgor pressure, form intimate intracellular connections, and modulate the binding sites and activity of growth factors, acting also as a local factor reservoir [7]. The composition dictates matrix stiffness and rigidity (affecting cell differentiation, migration, and proliferation), permeability (affecting nutrient diffusion to tissues and cell function), and cell-matrix interactions (affecting cell adhesion and proliferation) [8].

The ECM spatial arrangement, composition, and interaction with cells and growth factors are tissue- and function-specific. The epithelial ECM, for example, is minimal (only the basement membrane), on the contrary of connective tissues, characterized by an abundant ECM, while bone ECM consists mostly of collagen type I structural proteins, apatite mineral and noncollagenous proteins, such as osteocalcin, fibronectin, and vitronectin [9]. As a consequence, tissue peculiar characteristics, defining the unique biochemical, biomechanical, and structural profile, should be the goal to be achieved when defining a tissue engineered scaffold. For this aim several cues concur, which can be summarily classified into (i) internal ones, for example, the type and ratio of the “materials” that constitute that particular ECM, the hierarchical structure, the morphology and stiffness, and the signalling cascade between cells and extracellular environment, and (ii) external ones, being, for instance, dependent on the mechanical load exerted for a specific function, for example, muscle contractility/distensibility for food propelling within esophagus, bladder distension, or heart activity.

**Figure 2:**
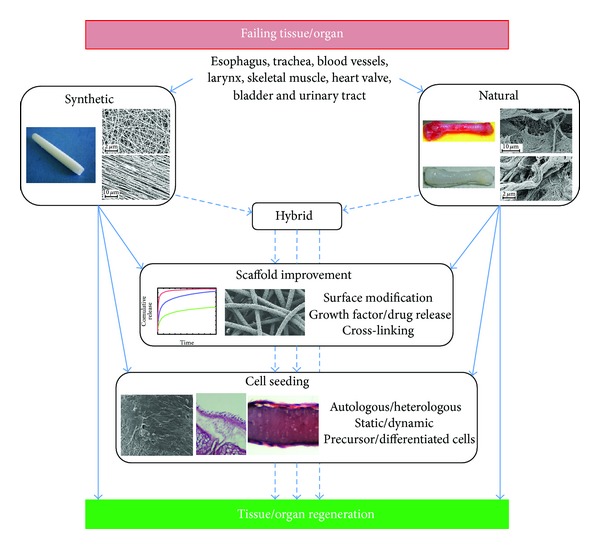
Strategies to develop a functional tissue engineered substitute for regeneration of failing tissues and organs. Synthetic, natural, or hybrid scaffolds can be treated to improve their features and performances, seeded with different cells types before implantation or directly* in vivo* implanted after the fabrication procedure.

The advantage of using natural scaffolds from innate organs allows to deal with substrates characterized by tissue-specific biochemistry (proteins and polysaccharides) and structural architecture, an intact and patent vasculature, and the presence of growth factors able to drive progenitor cell differentiation into organ-specific phenotypes [2]. In order to obtain a suitable ECM-like natural scaffold, the tissue should be preliminarily subjected to a decellularization process aimed to avoid any immunological response after implantation. However, it should be carefully considered that even if this strategy seems to be straightforward, the influence of the decellularization protocol adopted can harshly affect the final result leading to a structure that does not retain anymore the expected cues, specially the mechanical ones. Besides, the main limitation of native-derived scaffold is related to the shortage of cadaveric donor organs, which may significantly delay the obtainment of biological substitutes and increase patients' waiting time [10]. In order to overcome these limitations, an alternative route has been developed based on the use of synthetic scaffolds. Reasonably, the need for a donor is eliminated, and scaffold dimensions and shape can be tailored to fit the anatomy of the recipient due to intrinsic possibility to deal with a number of fabrication techniques and to select the most appropriate one [11]. Obviously, materials must be biocompatible, nontoxic, nonimmunogenic, and noncarcinogenic, should facilitate cellular adhesion, proliferation, organization, and differentiation, should be characterized by a suitable degradation rate and resist bacterial colonization [12]. It should be underlined that the ideal material, to be modelled into the specific organ to be regenerated, is still to come, as a single material can hardly recapitulate all the needed characteristics. An integrated strategy including possible postfabrication treatments and addition of fillers or drugs/growth factors for a subsequent release can be a desirable option.  Figure 2 summarizes possible strategies that can be considered for this aim, but at the same time this is only a macroscopic point of view since a detailed study of the tissue-specific ECM is necessary, especially referring to the intrinsic hierarchical nature. Clearly, the structure is strictly related to the final function and, in this regard, a nanoscale investigation of the characteristics composing each tissue can support the development of suitable scaffolds. As reported by Kim et al. [13], nanostructures in the human body can be classified into four categories dependent on the physiological environment: protective tissue (skin), mechanosensitive tissues (bone, ligament/tendon), electroactive tissues (neuron, skeletal muscle, and heart), and shear stress-sensitive tissue (blood vessel). This directly implies that the resulting mechanical properties play a crucial role in the expected tissue healing and regeneration, as rigidity can span in a wide range from few kPa to tens of GPa [14]. However, other features should be included when an ad hoc ECM-like scaffold has to be prepared. Metabolic activity is mainly based on a diffusion process of nutrients and removal of waste products and this is affected by porosity and permeability of the three-dimensional architecture; for instance, oxygen diffusion is limited to about 100 *μ*m from a blood vessel. In addition, cell-matrix interaction plays a pivotal role in the definition of the typical ECM dynamical environment, being related to its degradation that changes the local modulus, decreases the number of cell-matrix adhesion sites, and results in ECM fragments that may possess biological activity [8].

In this context, a critical review of the* in vivo* tissue engineering results collected so far can elucidate the role of an ECM-like scaffold, to be indented not only as a three-dimensional architecture to accommodate cells, but also as a complex and dynamic mixture of factors that concur to the expected target.

## 3. Tissue Engineered Orthotopic Approaches

In the last few years, there has been considerable progress in the clinical translation of tissue engineered organs, obtained using natural or synthetic scaffolds, repopulated with terminally differentiated cells or stem cells [15–21]. However, no gold standard strategy has been till now developed, possibly in relation to the fact that even “simple” organs, devoted only to transport functions (e.g., air, food, and liquids), represent a challenge.

The past strategies conducted to identify the ideal substitute for different organ/tissue will be here reported. As a guideline, Table 1 summarizes each biological district here considered in terms of (i) function, microstructure, histological features, and (ii) requirements that an ideal scaffold should provide. In order to understand which is the current real state of the art of the tissue engineering approach, the review will focus only on the most recent studies associated with relevant preclinical and clinical applications of natural and synthetic scaffolds.

## 4. Trachea

Being a relatively simple and hollow organ, the trachea was the ideal starting point for evaluating the possibility to obtain clinical relevant respiratory organ engineering. For more than 50 years, several approaches have been pursued to reconstruct the airways when conventional surgical approaches were unsuitable; however, the resulting clinical outcome was inconsistent, incomplete, and controversial [22, 23]. Most of the evaluated tissue engineering strategies resulted to be suitable only as patches for small airway repairs and were unable to resist collapse when used for whole long-segment (>6 cm) airway applications [24]. As a consequence, herein only the approaches focusing on tubular (for long circumferential defects) or trachea-like (bifurcated) scaffolds applied in preclinical and clinical studies will be reported.

### 4.1. Preclinical Studies

#### 4.1.1. Natural Approach

Different approaches have been tried to decellularize native airway; however a functional tissue engineered substitute has not been developed, due mainly to the decellularization protocol used, which, causing damage and/or disruption to ECM components, compromised the ability of the scaffold to provide mechanical support during the remodeling process [25–29]. Recently a detergent-enzymatic method (DEM), originally developed for the isolation of basement membranes from several tissues [30], has been modified and applied to animal airways. Bioengineered tracheal matrices with ultrastructure and physical properties similar to native tissue [31, 32], able to support* in vitro* adhesion of auricular chondrocytes and tracheal epithelial cells [33] and not to elicit any rejection response, were obtained [31]. Decellularized rabbit tracheas, obtained using DEM approach and preseeded both statically and dynamically with ovine amniotic MSCs, have been implanted into fetal lambs (9 to 12 tracheal rings) [34]. At retrieval, all the implants were characterized by variable degree of stenosis, but the engineered constructs showed full epithelialization, increased levels of α-elastin and collagen, and decreased GAG content pre- and postoperatively. The latter occurrence, as stated by the authors, was ascribed to the fact that cells were exposed to chondrogenic medium for 7 days before implantation, but GAG accumulation does not begin until the second or third week.

Porcine trachea matrices, decellularized by DEM, intraoperatively seeded with mononuclear cells (external surface) and epithelial cells (internal surface), and conditioned with a boosting factor treatment (transforming growth factor-*β*3, erythropoietin, and granulocyte colony stimulating factor), were tested in a porcine model. After 2 months, the implanted graft, even if not fully covered by cells, showed nearly native anatomic architecture and morphology [35].

#### 4.1.2. Synthetic Approach

Tubular high density polypropylene (20 mm in length and 8 mm in diameter) or PLA/PGA fiber (15 mm in length and 6 mm in diameter) scaffolds, preseeded with chondrocytes, allowed the formation of cartilaginous-like tissue with native-like biomechanical properties and mitigated the inflammatory reaction [36, 37]. Using both nasal septal chondrocytes and fibroblasts, PGA scaffolds, wrapped around silicon helical tube, have been implanted in mice (30 × 40 × 2 mm) or in sheep (50 mm in length and 20 mm in diameter) [38, 39]. Tissue morphology and composition resulted to be similar to native tracheal tissue, even if the implanted constructs collapsed and tracheomalacia was observed in sheep after anastomosis [38]. A straight* Dacron* prosthesis, reinforced with spiral-shaped polypropylene monofilaments, has been firstly implanted subcutaneously into rabbits, to allow the infiltration of blood vessels and connective tissue, and then was vertically opened and conditioned, on the inner surface, with tracheal epithelial cell sheets. The resulting bioartificial trachea was implanted (3 cm length): after 1 month a mature columnar epithelium was revealed in the interior graft portions, highlighting the importance of a functional epithelium lining the lumen of a prosthetic trachea [40].

Being a polymer with a slow degradation profile and mechanical characteristics potentially able to maintain the long-term patency, PCL has been widely used to develop tracheal prosthesis. Highly porous (pore size 10–40 *μ*m) hollow PCL tubular scaffolds, implanted in rabbits, were covered with epithelial cells, but animals died due to stenosis after 30 days from implantation. The same scaffolds, coated in the luminal surface with gelatin crosslinked with genipin, allowed a longer animal survival (43 days) and prevented the granulation tissue overgrowth. However, an epithelial lining was not revealed and the ingrowth of granulation tissue from the anastomotic sites into the lumen leads eventually to occlusion occurrences [41]. The same material was used to fabricate a scaffold (2 cm long) to be seeded with chondrocytes and bone marrow stem cells and implanted into rabbit abdominal wall for vascularization. Three weeks after tracheal replacement, the transplanted constructs retained good airway patency, did not collapse following removal of a silicone stent, and adequate vascularisation and muscular and epithelial regeneration were revealed in the neotrachea luminal surface. However, inflammatory changes and sputum accumulation occurred in the regions without epithelium-like tissue coating [42]. Tubular PGA fiber scaffolds, seeded with chondrocytes and implanted into sternohyoid muscle for 4 weeks, have been used, with or without muscle pedicle, to repair segmental defect of trachea with a silicon tube as stent (removed after 2 months). Six out of ten animals, implanted with vascularisation, survived over six months, while all the animals in the control group (without vascularization) died within two months after reconstruction, due to mucus impaction. 6 months after implantation, vascularised constructs retained structures and features of cartilage-like tissue and developed a continuous ciliated columnar epithelium layer, suggesting the importance of the prevascularization for the development of a suitable airway graft [43]. A copolymer of L-lactide and *ε*-caprolactone was synthesized to fabricate a sponge-like tubular structure (80% porosity; 20–100 *μ*m pore size; 6 cm length), reinforced by a woven fabric of PGA and coated with gelatin, to be implanted into sheep. After 9 months, only stent implanted substitutes (silicone stent, 7 cm length) had positive outcomes, even if a complete and spontaneous reconnection of the native trachea was not observed [44]. The same copolymer has been used to coat the luminal surface of a tubular scaffold, consisting of two collagen layers separated by a polypropylene framework and reinforced with 5 rings of polypropylene monofilament (30 mm long, 15 mm internal diameter) and implanted in dog left main bronchus [45]. After 14 days, the luminal surface resulted to be completely epithelialised with ciliated columnar and squamous epithelium, suggesting that the polymeric coating promoted an effective epithelialisation protecting the collagen layer.

Naito et al. [46] developed a tracheal prosthesis using a different approach: fibroblast and collagen hydrogels, mechanically supported by osteogenically induced MSCs in ring-shaped 3D-hydrogels, were implanted into rats (length 5-mm). The negative outcome (only three animals survived for 24 h and died the day after), due to strictures in the anastomotic regions, was mainly associated with the lack of an epithelial layer.

Regarding Y-shaped tubular scaffolds, a limited experimentation has been conducted till now. Sekine et al. [47] implanted into dogs Y-shaped scaffolds made of* Marlex* mesh (260 *μ*m pore size), reinforced with polypropylene spiral and coated with collagen. 14 out of 20 dogs died after experimentation due to obstruction of the main bronchus, omental necrosis, and air leakage. The same construct (60 mm long and 18 mm outer diameter) was tested as tracheobronchial bifurcation replacement: after 5 years, the prosthesis resulted to be completely incorporated by the host trachea and bronchus, neither stenosis nor dehiscence was observed, and a functional airway was revealed [48].

### 4.2. Clinical Studies

#### 4.2.1. Natural Approach

Using DEM approach, decellularized human tracheal matrices, characterized by structural and mechanical properties similar to native trachea, lack of immunogenicity, sufficient length for clinical application, containing proangiogenic factors, and supporting* in vivo* recellularization, have been developed [32, 49]. A bioengineered airway (7 cm long), dynamically preseeded with autologous epithelial respiratory cells and mesenchymal stem cell-derived chondrocytes, has been successfully used to replace left main bronchus (stenosed from tuberculosis) [18]. After 5 years, the patient is well, active, and, more importantly, has not shown neither an adverse immunological response nor serological signs of rejection, even without any immunosuppressive treatment [50]. Unfortunately, a recurrent cicatricial stenosis occurred at the native trachea closest to the transplanted trachea anastomosis, probably due to reduced mucosal blood flow, particularly when associated with lung inflammatory or infectious diseases.

In order to fast the obtainment of a suitable scaffold to be implanted and based on the experience performed with porcine model, decellularized human tracheas have been intraoperatively seeded with autologous bone marrow stromal cells, conditioned with growth and boosting factors and implanted to treat both benign (*n* = 5) and malignant (*n* = 3) airway diseases. The* in vivo* engineered transplanted tracheas resulted to be vascularised and lined with complete respiratory neomucosa; however a partial collapse of the most proximal part of the graft was observed in about 30% of patients [50]. This result could be probably due to an oxygen concentration gradient developing throughout the engineered trachea with consequent transmembrane cell migration from the outer (chondrocyte compartment) to the internal scaffold lumen (epithelial compartment). However, it is also possible that the decellularization process, slightly affecting the scaffold properties and surface topography, could have an effect on the long-term graft properties [10]. A short follow-up of 2 years verified the clinical outcome of this procedure [51]. The graft revascularised 1 week postoperatively, but an evidence of epithelium restoration was not observed before 1 year, which was verified after 15 months. The analysis of the decellularized scaffold showed the presence of 166 proteins relevant for regenerative medicine (e.g., angiogenesis and immunity), confirming the suitability of the proposed tissue engineering approach.

Recently, decellularized human trachea, repopulated with autologous stem cells, has been implanted into a 76-year-old patient with tracheal stenosis including the lower part of the larynx. After 23 days, the patient died due to cardiac arrest; however, the implanted construct resulted to be patent, also showing the presence of a squamous epithelium, neovascularization, muscular cells, serous glands, nerve fibers, and intact chondrocytes [52]. These clinical experiences demonstrated that functional tissue engineering natural-derived airway scaffolds can be obtained and are safe and promising. However, means to improve the biomechanical long-term stability of such grafts have to be developed before this technology can be translated into routine clinical practice.

#### 4.2.2. Synthetic Approach

A* Marlex* mesh tube (pore size 260 *μ*m; 50 mm long; 18, 20 or 24 mm inner diameter), covered with collagen sponge and reinforced with a supporting polypropylene ring and injected with autologous venous blood, was implanted into 4 patients affected by airway stenosis or cancer invasion [53]. During the postoperative observation period (8–34 months), a good epithelialisation was revealed in all the patients, and only in one case air leakage was observed.

A tracheobronchial graft made of POSS-PCU, both in casted form, for the cartilage “U” shaped rings, and in coagulated form, for the “connective” tracheal part, was implanted into a 36-year-old male patient affected by a recurrence of a primary tracheal mucoepidermoid carcinoma involving the distal trachea and both main bronchi [19]. Before implantation, the bioartificial scaffold was dynamically cellularized with autologous bone marrow mononuclear cells for 36 h. 1 week after operation a normal and patent airway was revealed, while biopsy samples showed the presence of necrotic connective tissue associated with fungi contamination and neovessels. After 2 months from transplantation, biopsy revealed large granulation areas associated with smooth epithelialisation and some organised vessels formation; bacterial or fungi contamination was not observed. An almost normal airway and improved lung function were assessed at 5 months.

## 5. Larynx

Attempts to construct an entire larynx have involved several approaches, and so far, tissue engineering has only been used for partial laryngeal reconstruction. The main problem is how to construct a bioartificial larynx with whole complex laryngeal framework, low immunogenicity, and dynamic function which requires combination of sphincter and breathing functions. Moreover, from a surgical point of view, the restoration of a laryngeal defect by means of a scaffold is challenging, because of vocal fold movement and the restriction to provide sufficient space for tissue regeneration [54].

### 5.1. Preclinical Studies

#### 5.1.1. Natural Approach

Tissue engineered cartilage grafts, obtained by seeding chondrocytes on hyaluronic acid (Hyalograft C)* via* a bioreactor, have been implanted in rabbits: despite no animals showed signs of respiratory distress, implanted grafts revealed marked signs of an unspecific foreign body reaction, leading to a complete degradation of the neocartilage and graft failure [55]. Cartilage sheets, obtained by seeded autologous cells on fibronectin-conditioned semipermeable polyester membrane* via* a bioreactor, have been implanted in rabbits for laryngotracheal reconstruction: the grafts showed no signs of degradation or inflammatory reaction, were covered with mucosal epithelium, but showed evidence of mechanical failure through migration and buckling [56].

Natural-derived scaffolds, obtained by tissue decellularizing, have been shown to allow a laryngeal repair superior to that observed using control standard procedure [57–59]. The regeneration of thyroid cartilage, epithelium, connective tissue, glandular structures, and some skeletal muscles has been obtained in dog by implanting porcine decellularized urinary bladder matrix [57]. Rabbit tracheas have been decellularized using a perfusion decellularization protocol to simultaneously reach all parts of the larynx and to create a low-immune whole-larynx scaffold comprising decellularized laryngeal muscles, a reserved decellularized matrix, and an integrated cartilage framework. The grafts, reseeded with MSCs and implanted in rabbits, allowed the regeneration of muscle bundles and vessels, even if a severe immunological reaction was observed [60]. A recent study evaluated the regenerative effects of acellular porcine urinary bladder matrix on hemilarynx, using a canine model. After one month, all animals showed good reepithelialization with minimum complication, while after 6 months postoperatively, cartilaginous structures, normal (or near normal) phonation threshold pressure, and mucosal wave amplitude were assessed. Even if the regenerated vocal fold mucosa resulted to be scarred, the acellular urinary bladder scaffold can be regarded as a potential means for a functional tissue regeneration of the hemilarynx [61].

#### 5.1.2. Synthetic Approach

*Marlex* mesh (pore size of 260 *μ*m), reinforced with a polypropylene supporting ring, coated with collagen, and preclotted with arterial blood, has been used in dogs for the treatment of subglottic stenosis: the scaffold resulted to be well integrated and covered by epithelial cells; however the presence of granulation tissue and mesh exposure were reported [62]. The same synthetic scaffold has then been developed based on the replication of the luminal canine larynx and was used to perform a hemilaryngectomy. The scaffold has been preclotted with a mixture of peripheral blood and bone marrow-derived stromal cells and implanted in dogs: soft tissue regeneration (after 8 days) and the presence of mucosal cells (after 3 weeks) were observed [63]. Recently,* Marlex* mesh, coated with collagen and wrapped with autologous fascia, resulted to be a viable alternative for the regeneration of laryngeal defects. However, scar-like tissue with consequent reduction of the treated vocal fold, exposure, or dislocation of the mesh was revealed, suggesting that additional approaches are required to regenerate a normal and functional vocal fold [54].

### 5.2. Clinical Studies

#### 5.2.1. Natural Approach

A free-tissue transfer and an autologous tracheal segment have been successfully used for partial laryngeal replacement preserving one muscle-nerve-joint unit, providing vocal and sphincter functions [64]. Good breathing results were reported, while, due to the lack of a truly laryngeal architecture, voice and swallowing remain to date suboptimal. The availability of substitutes displaying equivalent anatomical, physiological, and biomechanical properties compared to normal human larynxes would provide the right, complex architecture, and dynamics for normal voice production and sphincter action.

#### 5.2.2. Synthetic Approach

Considering the efficacy evaluated in preclinical studies,* Marlex* meshes, coated with collagen and injected with autologous venous blood, have been implanted into 4 patients affected by airway stenosis (*n* = 1 subglottis stenosis) or cancer invasion (*n* = 3 thyroid cancers) [53]. A good epithelialisation occurred in all patients during the postoperative observation period, and air leak was revealed in only one case. These promising results demonstrate the ability to regenerate cricoid cartilage using scaffolds composed of polypropylene and collagen sponge in clinical applications.

## 6. Esophagus

Esophageal substitution is generally required in presence of several pathological conditions, for example, esophageal atresia, acquired constriction esophagitis, esophagotomy, or cancer, becoming the world's sixth leading cause of death [65, 66]. Surgical resection is regarded as a standard treatment in the early stage of the disease, involving gastrointestinal segments as potential substitutes to repair the esophageal defect. However, this procedure is not free of drawbacks, as the incidence of complications is relatively high, and the mortality rate is up to 4% [67]. In this regard, the development of a suitable alternative according to the tissue engineering approach might represent the desired option, as the following reported studies suggest.

To the best of our knowledge, no clinical studies have been till now performed following the synthetic route, highlighting, once again, the complexity of designing a reliable scaffold even for a tubular organ mainly characterized by a transport function.

### 6.1. Preclinical Studies

#### 6.1.1. Natural Approach

Natural-derived matrices have been used both as esophageal patches and as tubular scaffolds. Porcine-derived, xenogeneic ECM obtained from SIS, seeded with autologous oral mucosal epithelial cells, has been used as patch to repair an esophageal defect (5 cm length and 2.5 cm width) in a canine model [68]. The seeded scaffolds showed a better healing process compared to the control group (unseeded scaffolds): a complete reepithelialization was observed 4 weeks after surgery, while extension of the muscular bundles and appearance of island muscle cells in the connective tissue were revealed after 8 weeks (muscle cells generation was not observed in the control group). Patches made of porcine SIS or urinary bladder submucosa were characterized by a positive outcome when implanted into adult dogs to repair a defect of approximately 5 cm in length and encompassing 40%–50% of the circumference of the esophagus [69]. The naturally derived material resorbed within 2 months, and the presence of organized skeletal muscle bundles and of a confluent squamous epithelium was observed. Following a similar approach, acellular porcine SIS, seeded with bone marrow MSCs, has been used to replace a section of canine cervical esophagus (5 cm in length and 50% in circumference). After 12 weeks from surgery, a complete construct reepithelialization, revascularization, and muscular regeneration were revealed, with almost no inflammation signs [70]. The use of matrices derived by SIS gave similar promising results for the treatment of esophageal semicircumferential defects in rats [71], used as an animal model also to test the gastric acellular matrix, an alternative naturally derived material [65]. In this latter case, rat gastric acellular matrix was prepared to recover a defect (5 mm length and 3-4 mm width) in the abdominal esophagus of the same animal model. Two weeks after implantation, the regeneration of keratinized stratified squamous mucosa was assessed, but no ingrowth of the inner or outer muscle layer was detected after 18 months. Interestingly, a decellularized esophagus was tested as a potential scaffold to repair an esophageal defect, which seems to be a logical approach due to the intrinsic similarity of the “artificial” patch-organ microstructures [72]. A 2 cm defect in the tunica muscularis of the thoracic pig esophagus was covered with an esophageal homologous matrix, seeded or not with autologous SMCs. At 3 weeks from surgery, both patches were infiltrated by mononuclear cells and fibroblasts without signs of rejection, even if unseeded scaffolds showed a severe inflammatory response and were negative for *α*-smooth muscle actin immunostaining. On the contrary, seeded implants were characterized by SMC ingrowth, with an early organization into small fascicules.

A tubular scaffold for esophageal regeneration was evaluated by Badylak et al. [73]. Porcine urinary bladder matrix was shaped into a tubular construct to be used in conjunction or not with muscle tissue to recover a canine esophageal circumferential resection of 5 cm. For this aim, four groups were considered: ECM scaffold alone, muscle tissue alone, and ECM plus either a partial (30%) or complete (100%) covering with muscle tissue. Animals of the first two groups developed severe strictures within the 3 weeks, while a constructive remodelling was verified in the last two groups. Also matrices derived from SIS have been tested as tubular scaffolds and implanted in the cervical esophagus of piglets (about 4 cm in length) [74]. However, implanted matrices lead to a high rate of esophageal stenosis, suggesting that, dealing with a decellularized ECM could not assure the desired results, even if a similar microstructure is provided to the surrounding tissue. More specifically, dynamically decellularized rat oesophagi were seeded with allogeneic mesenchymal stromal cells which spontaneously differentiated into epithelial- and muscle-like cells. The reseeded scaffolds were then implanted to orthotopically replace the entire cervical oesophagus in immunocompetent rats. All animals survived the 14-day study period, with patent and functional grafts, and gained significantly more weight than sham-operated animals. At retrieval, explanted grafts showed regeneration of all the major cell and tissue components of the oesophagus, including functional epithelium, muscle fibres, nerves, and vasculature [75].

#### 6.1.2. Synthetic Approach

To repair semicircular esophageal defects (0.5 × 1 cm), both absorbable (*Polyglactin 910*, Vicryl) and nonabsorbable (polyvinylidene fluoride) meshes were implanted in rabbits [76]. Mucosal regeneration was observed, with the nonabsorbable meshes leading to better results. The absorbable scaffolds lead indeed to early degradation with consequent ulceration, abscess formation, and diffuse inflammation. To improve tissue regeneration, the implantation of cell seeded scaffold has been evaluated. PGA/PLA scaffolds, seeded with rat adipose smooth muscle-like cells, were implanted in rats in order to repair a defect (3 mm width; 5 mm length) created in the abdominal esophagus [77]. A complete reepithelialisation of the esophageal lumen was observed after 10 weeks, while muscularis layer regeneration was detected at 16 weeks. PCL meshes, seeded with smooth muscle and epithelial cells, have been implanted in the abdominal part of rabbit esophagus (0.6 × 1 cm). Fifteen rabbits survived the trial period (30 days), and 6 out of 20 animals had no complications. The synthetic mesh was almost completely degraded and replaced by a layered continuum of epithelium and SMCs, organized in varying degrees [78].

In order to provide a more complex and anatomical-like engineered substitute, a typical surgical approach to promote esophagus scaffold-guided regeneration consists in wrapping a selected material around a tube to impart and retain the tubular shape after implantation; the tube is then removed and the resulting scaffold should act as a functional device. This strategy was followed by Takimoto et al. [79]: a 5 mm thick freeze-dried collagen sponge was wrapped around a silicone tube and implanted into dogs to recover a 10 cm defect. Two animals died after tube removal (6 weeks), while after 6 months a completely epithelialized luminal surface, normal esophagus glands, and immature muscle tissue were revealed. Similarly, a PLA : PCL (50 : 50) mat, reinforced with PGA fibers, supported by a nasopharyngeal airway tube, was used to repair an esophageal oval-shaped defect (4 × 2 cm) in a pig model [80]. The tube was used only as temporarily support and was sutured to the scaffold with short-term sutures in order to allow the tube falling off into the intestine. After 4 weeks squamous epithelium was regenerated, and after 12 weeks the muscular layer was similar to the native tissue. However, the main limit of this strategy is the dislodgment of the stent which might cause bowel obstruction. A scaffold, made of nonwoven PGA used as a substrate for human amniotic membrane seeded with canine oral keratinocytes and fibroblasts, rolled around a polypropylene tube (3 cm in length and 2 cm in diameter) and previously implanted in dog abdomen for 3 weeks, was used to replace a 3 cm esophageal resection [81]. Unseeded grafts developed strictures, with consequent complete esophageal obstruction, while seeded ones showed the regeneration of squamous epithelium, muscularis mucosa, and smooth muscle tissue and a good distensibility. However, the presence of esophageal glands and peristalsis was not detected.

A more sophisticated approach considered 2 mm thick nonwoven tubular PGA (15 *μ*m fiber diameter) scaffolds (1 cm length; 0.5 cm outer diameter, 0.2 cm inner diameter), seeded with neonatal or adult rat esophagus organoid units (mesenchymal cores surrounded by epithelial cells), sealed with poly-L-lactic acid, and implanted in syngeneic hosts [82]. Esophageal architecture with keratinized squamous epithelium and an actin-positive muscularis was revealed and no signs of deterioration were observed for 42 days.

### 6.2. Clinical Studies

#### 6.2.1. Natural Approach

To treat a cervical esophageal perforation in an 82-year-old man, Clough et al. [83] implanted an acellular matrix (Surgisis) derived from porcine SIS. A patch of 5 × 3 cm was used to repair the defect and after 6 days from surgery no leakage was detected, allowing commencing oral intake. At 4 weeks, the healing process was confirmed displaying a normal calibre esophagus; the patient was discharged after 61 days and remains well 4 months postoperatively, even if a normal swallowing was not reestablished. The same scaffold was also used to treat Barrett's esophagus with high-grade dysplasia and mucosal adenocarcinoma by means of a minimally invasive endoscopic procedure [66]. Five male patients (average age 62.0 ± 5.3) were subjected to circumferential, long segment sleeve resection of mucosa and submucosa and placement of the biological scaffold, secured into position with a radially expanding stent, resulting in gentle compression against the muscularis externa, removed after 9–18 days postoperatively. During the follow-up (4–24 months) a progressive tissue restoration was observed with the formation of squamous epithelium.

## 7. Heart Valves

Currently, the failure of pathological heart valves is surgically treated by means of an artificial prosthesis, either mechanical or biological, the latter being a porcine-derived heart valve or made of bovine pericardium. However, none of these two models is free of drawbacks that can concur to limit the performance of the implanted valve. Due to the nature of the materials and the nonphysiologic hemodynamics, mechanical valves require a lifelong anticoagulant therapy that can expose the recipient to hemorrhagic events. On the other hand, biologic valves, due to a degenerative calcific process, are characterized by a limited temporal functionality [84]. A novel alternative is therefore desirable and tissue engineering might furnish the solution to the problem. Reasonably, this is not expected in the very next future since a reliable safe heart valve has not been developed yet and a large number of research groups have been focused on different aspects of the issue and, as a consequence, multiple routes have been traced. It is interesting to notice that most of the pivotal studies focusing on the assessment of a functional tissue engineered heart valve prosthesis are based on the use of naturally derived decellularized heart valves, and an entire synthetic polymeric heart valve tested in an* in vivo* model is still to come. This might be due to the peculiar features of this anatomical district, implying the development of a proper strategy to fabricate a valid substitute that closely matches the natural one. Several synthetic approaches can be cited that moved along this route, but the related evaluation has been mainly conducted* in vitro* [85]. Therefore, a decellularized scaffold seems to be a suitable choice at present, since it offers a morphology, binding sites, and three-dimensional architecture that is naturally designed for the expected function.

### 7.1. Preclinical Studies

#### 7.1.1. Natural Approach

Decellularized allogenic pulmonary valve conduits were orthotopically implanted into a sheep model after being reseeded with autologous myofibroblasts and endothelial cells [86]. Transplantation of unseeded acellular valves (control group) leads to only a minimal immigration of myofibroblasts without matrix reorganization or procollagen synthesis, but both cell seeded or unseeded valves were characterized by a confluent endothelial cell lining after 3 months. The latter result, according to the authors, could be influenced by the animal model used in the study and may be more limited in human beings without preseeding. A heart valve completely made of fibrin was proposed by Flanagan et al. [87]. Fibrin-based valves were cast in customized molds by mixing fibrinogen solution, saline solution containing carotid artery ovine SMCs and fibroblasts, and calcium chloride. Polymerization was started by adding thrombin solution. After being conditioned in a bioreactor for 28 days, valves were subsequently implanted into cell-donor animals in the pulmonary trunk, leaving intact the native pulmonary valve to prevent acute volume load of the right ventricle due to a possible incompetence of tissue engineered valves. At 3 months after surgery, all valve conduits were patent, covered by a confluent layer of endothelial-like cells with viable interstitial-like cells throughout the entire valve thickness. Moreover, the production of autologous collagen and ECM proteins, replacing the fibrin, was also assessed.

The* in vivo* spontaneous recellularization of acellular matrix was evaluated by implanting decellularized porcine aortic valves in the descending thoracic aorta of lambs and providing subcutaneous injections of G-CSF, as boosting factor to mobilize early bone marrow progenitor cells [88]. However, this treatment did not improve scaffold recolonization but, rather, induced accelerated valve deterioration, enhanced inflammatory cell infiltration, and neovessel formation in the adventitia, myointimal proliferation, and calcifications, both in leaflets and the aortic wall. The* in vivo* reendothelialization of decellularized ovine aortic valve allografts, implanted as an aortic root in lambs, has been evaluated also by Baraki et al. [89]. At retrieval (3 and 9 months postoperatively), partial luminal endothelialisation, neovasculogenesis at the adventitial side, trivial regurgitation, and normal morphology with no signs of graft dilatation, degeneration, or rejection were reported. On the contrary, marked calcification/degeneration and advanced valve insufficiency were revealed in control valves group (fresh native ovine aortic valve conduits). In order to improve graft recellularization, cells have been seeded on natural-derived scaffolds before implantation. Canine myofibroblasts and endothelial cells have been seeded on acellular porcine aortic valve leaflets, which, after a 7-day incubation period, have been implanted into the lumens of canine abdominal aortas [90]. After 70 days from surgery, histochemistry confirmed that myofibroblasts grew within the matrix, while valve leaflets were partially covered by endothelial cells. In addition, no evidence of calcification was observed. A similar technical approach has been presented by Kim et al. [91]: canine endothelial and myofibroblast cells, derived from allogenic BMCs, have been seeded on decellularized porcine heart valves, before being implanted into canine abdominal aorta and pulmonary valve. At retrieval (1 and 3 weeks postoperatively), grafts showed a normal morphology, but an incomplete endothelialization and regeneration were observed, due to a nonhomogenous cell seeding and the subsequent detachment after implantation. Cell seeding before implantation represents a crucial issue to be addressed. It is well known that, based on the complete tissue engineering paradigm, a proper tissue engineered scaffold is the concurrent result of an effective cell-matrix interaction. Tudorache et al. [92] further proved this point dealing with an ovine decellularized aortic valve conduit, reseeded with autologous endothelial cells, conditioned* in vitro* into a bioreactor, and then implanted in orthotopic position into sheep (cryopreserved valves were used as control). The proposed constructs were not affected by valvular insufficiency, stenosis, and cusp thickening, differently from the control group. In particular, the degeneration of the cryopreserved valves was related to the presence of allogenic cells and disorganization of ECM components as a result of storage and mechanical stress.

On the other hand, cell seeding can be considered a time-consuming procedure and can be overcome planning a specific strategy. In this regard, decellularized porcine pulmonary valves, conjugated with CD133 antibody, were transplanted in the pulmonary position into sheep, while unconjugated and autologous endothelial cell-reseeded valves were used as control [93]. This study proved that a more cell-rich valve can be obtained: the production of matrix proteins improved, as collagen and GAGs increased from 1 to 3 months postoperatively, and the biomechanical properties were similar to those of a normal valve, compared to the control cases. However the functional remodelling was not assessed.

#### 7.1.2. Synthetic Approach

An interesting use of resorbable polymers for tissue engineering heart valves was proposed by Wu et al. [94] fabricating a hybrid heart valve made of decellularized porcine aortic valves coated with PHBHHx, which belongs to polyhydroxyalkanoates, a class of polymers of microbial origin. The resulting scaffold was implanted in pulmonary position in sheep and, even if the polymeric surface covered the ECM beneath, a positive outcome (16 weeks postoperatively) was assessed as it contributed to prevent the activation of thrombogenic matrix components, protected ECM from the potential harmful influence of host fluids, and supported repopulation with recipient's myofibroblasts and endothelial cells. An example of the performance of a synthetic valve conduit was presented by Gottlieb et al. [95], assembling nonwoven sheets containing PGA and PLA fibers (50% : 50%). Firstly, grafts have been seeded with MSCs from neonatal sheep bone marrow, then cultured for 1 month, and finally implanted in pulmonary position, after having excised the native valve cusps and 1-2 cm main pulmonary artery segment. The diameter of the implanted valve conduits remained unchanged for 20 weeks; however cusp dimensions decreased leading to pulmonary regurgitation after 6 weeks. In order to improve the clinical outcome of congenital cardiac defects, prenatal heart valve interventions can be a valuable strategy [96]. Trileaflet heart valve scaffold, made of nonwoven PGA meshes, integrated into radially self-expandable nitinol stents, and coated with poly-4-hydroxybutyrate, was seeded with ovine amniotic fluid cells and implanted orthotopically into the pulmonary position using an in-utero closed-heart hybrid approach. Tissue engineered valves showed intact and mobile leaflets with no thrombus formation or impairment of substitute integrity. The same materials and the same valve design were used for pulmonary valve substitution in nonhuman primates, either considering autologous bone marrow-derived mononuclear cell seeding [97] and the decellularization approach [98]. In the latter case, scaffolds were firstly seeded with human fibroblasts, dynamically cultured for 4 weeks, and decellularized to be implanted into baboons in orthotopic pulmonary valve position by means of an antegrade transapical approach. Postoperatively, the implanted valves were characterized by a mild-moderate insufficiency, leaflet shortening, and rapid cellular repopulation, compared to decellularized native human heart valve control.

## 8. Vascular System

The most common treatment for cardiovascular diseases is the use of an autologous vascular graft, retrieved from the internal mammary arteries and saphenous veins; however this approach can be limited by patient's age and pathology [99]. Endothelium restoration/regeneration is one of the major aims of vascular tissue engineering, since it plays a crucial role in vascular biology regulating permeability, inflammation, thrombosis, and fibrinolysis [100, 101]. Endothelial growth can be supported* in vivo* with appropriate scaffolds with mechanical stability and remodelling potential. Several scaffolds have been developed for vascular tissue engineering; among these, it is possible to distinguish between three categories that have been* in vivo *tested: decellularized matrices, self-assembling vessels, and electrospun synthetic scaffolds.

### 8.1. Preclinical Studies

#### 8.1.1. Natural Approach

In an early study, porcine iliac blood vessels were decellularized with 1% TritonX-100 and 0.1% ammonium hydroxide for 72 h, seeded with EPCs, and tested in a sheep common carotid artery substitution model [102]. EPC-seeded grafts remained patent for 130 days, whereas nonseeded grafts occluded within 15 days. The seeded grafts exhibited contractile activity and nitric-oxide-mediated vascular relaxation. The study did not show analysis of dilation, thrombogenesis, or intima hyperplasia. The same decellularization protocol was used also in other works to fabricate acellular canine and porcine carotid arteries [103, 104]. Canine matrices were seeded with bone marrow derived cells, differentiated toward smooth muscle and endothelial cells, and implanted in cell donor dogs in a carotid artery interposition model [103]. The acellular scaffold was primarily composed of elastin and collagen and exhibited porous structure. Cell seeded vascular grafts remained patent for up to 8 weeks, whereas unseeded grafts occluded within 2 weeks. Matrix remodelling was evident with maintenance of the 3 layers and the graft demonstrated appropriate mechanical strength to endure forces exerted by sutures during surgery. Longer time points (4 months) in a similar approach in pig showed no structural failures, aneurysms, or infectious complications [104].

Decellularized veins have been developed using also other detergents, such as SDS: decellularized canine external jugular veins have been transplanted in a carotid interposition model in dogs [105]. No graft deterioration, in terms of rupture, anastomotic complication, or dilation, was observed 8 weeks after implantation, with minimal inflammation, transmural repopulation of vascular cells, and a compact fibrin layer formed along the lumen. Unfortunately, no longer time points were analysed. Decellularized porcine saphenous arteries have been implanted into rabbit carotid arteries: after 3 months grafts showed 60% patency rates, regeneration of vascular elements, with no aneurysm and intimal hyperplasia events, suggesting their potential as small-diameter grafts [106]. Decellularized equine carotid arteries, coated with a matricellular protein (CCN1) and implanted as cervical arteriovenous shunts, showed, 14 weeks after implantation, smooth muscle regeneration, complete endothelialisation, and immunologic tolerance, suggesting CCN1 coating as a promising tool for generation of bioartificial vascular prostheses [107].

Acellular human umbilical cord arteries have also been evaluated* in vivo* as vascular tissue engineered graft [108]. These vessels, decellularized with CHAPS buffer and SDS buffer for 4 days, were incubated for 2 days in endothelial growth media-2. The acellular arteries retained the majority of the ECM components (collagen and elastin) and similar mechanical properties (maximum burst pressure and maximum modulus) to the native vessels. To evaluate the mechanical strength of decellularized umbilical arteries* in vivo*, vessels were implanted into nude rats as abdominal aorta interposition grafts. The scaffolds were mechanically robust* in vivo* for 8 weeks with no dilation or aneurysm formation, but the absence of a cellular compartment in the construct seemed to cause occlusions within 24 h after implantation and various levels of thrombosis. Furthermore, no cell infiltration was observed within the scaffold, probably due to the short time observation period.

Cell self-assembling scaffolds are composed by cell-derived ECM sheets developed* in vitro* to produce vascular grafts of arbitrary lengths. Cells are normally autologous in order to obtain a total biocompatible graft [109–113]. A pioneering study has been published in 1999 where smooth muscle and endothelial cells, derived from a biopsy of a vascular tissue, were cultured for 8 weeks in a pulsatile perfusion system [109]. These engineered vessels were composed of alive and functional cells, secreting ECM, mainly collagen. The grafts showed rupture strength greater than native human saphenous veins and contractile responses to pharmacological agents. Tissue engineered arteries were implanted in swine into the right saphenous artery, showing patency up to 24 days after transplantation, without evidence of stenosis or dilatation. The xenograft had also unchanged contractile responses to prostaglandin and there was no evidence of bleeding at the anastomoses or mechanical breakdown at explantation. A similar approach used a combination of human vascular SMC-derived sheets and human fibroblasts sheets to provide the adventitia. After maturation, the tubular support was removed and endothelial cells were seeded in the lumen [110]. Histological analysis revealed well-defined tissues (intima, media, and adventitia) and a complex and naturally organized ECM, although single components were not quantified and characterized. Short-term grafting experiment in a canine model demonstrated good handling and suturability characteristics, mechanical stability, and blood compatibility. Human fibroblast sheet-derived vascular grafts were also tested in nude rats (long-term study) and primates (for up to 8 weeks), showing physiological mechanical strength and positive scaffold remodelling with activation of resident cells [111]. Another similar study confirmed the applicability of this technique using MSC-derived sheets organised to produce a vascular graft [113]. Here, the constructs were implanted into common carotid artery defects of rabbits and analysed after 4 weeks, evidencing excellent patency and good integration with the native vessel. No stenosis, thrombus formation, or inflation occurred in this short time point.

Alternatively, allogeneic cells can be cultured on degrading tubular scaffolds and then decellularized to eliminate the cellular compartment maintaining the cell-secreted ECM as an acellular scaffold. This hybrid approach has been proposed to fabricate human allogeneic, porcine or canine smooth muscle cell-derived constructs, starting from tubular PGA scaffolds [114, 115]. The acellular grafts, subsequently seeded with autologous cells (endothelial or endothelial progenitor cells) on the lumen, have been then transplanted* in vivo* in porcine, baboon, or canine vessel transposition models. Scaffolds demonstrated excellent patency for up to 1 year, resistance to dilatation, calcification, and intima hyperplasia. Infiltration of smooth muscle positive cells, endothelial cells, and elastin formation were observed near anastomoses. Endothelial cell-seeded scaffolds showed capacity to better maintain patency after* in vivo* implantation with respect to synthetic constructs [115]. The combination of natural and synthetic materials was also assessed using decellularized umbilical arteries, coated with polyelectrolyte multilayers (3.5 bilayers of poly(styrene sulfonate)/poly(allylamine hydrochloride)), seeded with endothelial cells, and cultured* in vitro*. The constructs were successfully implanted as rabbit carotid substitute, demonstrating that preconditioning is a crucial factor for graft patency [116].

#### 8.1.2. Synthetic Approach

PCL micro- and nanofiber-based vascular grafts were evaluated in a rat abdominal aorta replacement model for up to 18 months [117]. No dilatation or thrombosis and limited intimal hyperplasia were shown together with endothelialisation, cell invasion, and neovascularisation of the scaffold. Neverthless, after 18 months, the graft was remodeled, even if blood capillaries were not present and a calcification process was detected in the layers. PCL showed poor compliance before transplantation and did not improve throughout the time points. The same authors implanted in a rat model as an aortic replacement a PCL vascular graft with increased hydrophilicity (obtained by plasma treatment). After 3 weeks, a recellularized graft was revealed, suggesting that plasma treatment could be a strategy to easily increase the biocompatibility of a scaffold and accelerate tissue regeneration without compromising mechanical strength [118]. When compared to ePTFE electrospun grafts, transplanted in the same rat model, PCL scaffolds showed significantly better endothelial coverage, macrophage and fibroblast ingrowth, ECM deposition and angiogenesis, and no stenotic lesions up to 24 weeks [119]. In these experiments, however, chondroid metaplasia occurred after 6 weeks and was then replaced by calcification. A combination of PCL and collagen was used to improve cell adhesion and growth into the graft in a rabbit arterial bypass model [120]. This work indicates that the construct supported* in vitro* cell adherence and maintained structural integrity and patency over 1 month of implantation, but no cell invasion or evident endothelialisation was shown after this short time point. A scaffold composed of *ε*-caprolactone and lactic acid [P(CL/LA)], 50 : 50 ratio, was seeded with bone marrow cells and implanted in the inferior vena cava of dogs [121]. Analyses were more focused on cell proliferation and differentiation after 8 weeks after implantation, but histology showed graft remodelling with new ECM deposition. Natural and synthetic polymers were blended into an electrospun scaffold composed of chitosan and PCL to combine the bioactive functions of the first one (biocompatibility, low toxicity, and antibacterial properties) with the good mechanical properties of latter one [122]. The scaffold was seeded with autologous outgrowth endothelial cells harvested from canine peripheral blood and expanded* in vitro* and then implanted into carotid arteries of cell-donor dogs for 3 months. Chitosan/PCL scaffolds were characterized by nanofiber average diameter (550 ± 120 nm), porosity (more than 80%), and tensile strength proper for vascular tissue engineering. Seeded scaffolds remained patent as compared with unseeded grafts 3 months after implantation, with tissue remodelling (presence of collagen and elastin) and regeneration of a functional endothelium. Furthermore, biomechanical properties were close to native carotid arteries when grafts were collected 3 months after transplantation, with no blood leaking or deformation. In Hoerstrup et al. [123], PGA meshes were coated with a thin layer of poly-4-hydroxybutyrate, seeded with myofibroblasts and endothelial cells, and surgically implanted as main pulmonary artery replacement in lambs for up to 100 weeks. The animals more than doubled their body weight during the 2-year period. Regular echocardiography and angiography showed good functional performance and absence of thrombus, calcification, stenosis, suture dehiscence, or aneurysm. There was a significant increase in diameter by 30% and length by 45%, cellular engraftment, and new ECM deposition in all the groups, but the mechanical profiles of the graft were lower than native pulmonary arteries.

Cell-free PGA vascular graft, reinforced with P(CL/LA) monofilaments on the outer surface to maintain the shape for the initial crucial period following implantation, was implanted in a canine model of substitution of inferior vena cava (24 months) and pulmonary artery (12 months) [124, 125]. In both cases, histological examinations revealed a well-formed vessel-like vasculature without calcification and similarities to native vessels in terms of patency and biomechanical properties. The slower degradation rate of P(CL/LA) guaranteed constant construct elasticity during the tissue-remodelling process, resulting in prevention of stenosis and endothelialisation improvement. However, vascular SMCs were not well-developed 12 months after implantation in the pulmonary artery model [125]. Recently, the authors demonstrated that after 24 months, this biodegradable scaffold can gradually regenerate and develop into a mature vessel characterized by histological and biochemical properties similar to the native tissues [126]. The same combination of PGA and P(CL/LA) (80% porosity; 20–50 *μ*m diameter) has been used with bone marrow derived cell seeding in an inferior vena cava substitution in dogs, showing higher remodelling and endothelial and smooth muscle cell invasion of the graft with respect to unseeded scaffolds, even though the latest time point was 4 weeks [127]. Tissue engineered PGA and P(CA/LA) scaffolds, seeded with bone marrow cells and implanted in an immune competent mouse model, demonstrated that this type of constructs, functionalized by mobilizing resident cells, allows the activation of the innate healing process, more than an active participation of the cells delivered concurrently with the scaffold [128–130].

Reinforced multiple layer scaffolds, created using polymers, like poly(ester urethane)urea, and obtained by thermally induced phase separation and electrospinning (for the outer layer) procedure, have shown higher resistance with lower mechanical failure and dilatation [131]. Multilayer tubular conduits, made of collagen fiber networks and elastin-like protein polymers, characterized by collagen ultrastructure (internal diameters: 1 and 4 mm) and mechanical properties similar to native blood vessels with limited platelet adhesion, were implanted in a rat aortic interposition model. After 14 days after implantation, grafts appeared patent with minimal adhesive response and with a limited early inflammatory response, suggesting that engineered collagen-elastin composites could represent a promising strategy for fabricating synthetic tissues with defined ECM content, composition, and architecture [132].

In order to accelerate construct remodelling and integration, a fast degrading elastomer, poly(glycerol sebacate), has been recently used in rat abdominal aorta substitution model [133]. The graft, coated with heparin to enhance thromboresistance, showed mechanical properties able to promote vascular cell differentiation and avoid stress shielding, being also characterized by high porosity with interconnected pores. Three months after transplantation, these cell-free grafts resembled native arteries in terms of pulsation, endothelium and smooth muscle layer development, expression of elastin, collagen and GAGs, and compliant mechanical properties. Scaffold integration and remodelling were almost complete after 3 months, suggesting that rapid graft degradation may be helpful for cell infiltration, positive inflammation, and new matrix production.

### 8.2. Clinical Studies

#### 8.2.1. Natural Approach

Autologous human fibroblast and endothelial cell derived grafts were evaluated in a clinical trial including ten patients implanted with a completely biological and autologous tissue engineered vascular graft [112]. Self-assembling vessels, giving natural cell-produced ECM with established mechanical properties and* in vivo* applicability, showed promising results in early clinical applications. However, extensive* in vitro* culture and high costs are required [134].

## 9. Kidney

Even if kidney is the most commonly transplanted organ, its availability is limited [135]. Hemodialysis represents a real contribution to the survival of patients with end-stage renal disease, but transplantation is the only available curative treatment [136]. Clearly, the ultrastructure and function of this organ pose a significant challenge for the definition of a valuable tissue engineered substitutes. This concurs to explain the degree of the development of a viable alternative in terms of materials selected and* in vivo* experimentation. Ideally, a tissue engineered kidney should be able to replace all the functions, including endocrine and metabolic activities and removal of uremic protein-bound waste products [137]. The attention was therefore focused on those studies dealing with the whole organ, treated with specific protocols aimed to preserve the anatomical integrity and assess the potential to restore its peculiar physiology.

### 9.1. Preclinical Studies

#### 9.1.1. Natural Approach

Using SDS, Orlando et al. [138] demonstrated an effective decellularization of porcine kidneys, preserving organ structure (including vascular network) and function. A particular care was adopted in the selection of the most suitable decellularization protocol. SDS may disrupt the native tissue architecture and damage some ECM components, but it was preferred to an enzymatic method, due to the adverse effect that enzymes may exert on digestion-sensitive molecules in the ECM, or to snap freezing because exposure to extremely low temperatures may alter the three-dimensional architecture of the scaffolds. The obtained decellularized kidney scaffolds were implanted for two weeks in pigs, matched for age and weight to the scaffold donors. The surgical procedure was technically feasible as the mechanical properties of the vasculature deprived of the endothelial layer supported the surgical reconnection of the vessel stumps of the scaffold to the recipient's aorta and vena cava. This study showed that the organ was reperfused and the scaffold implantation was well tolerated with no adverse reaction. However, according to the authors, the lack of endothelium elicited the formation of massive thrombi within the renal artery and vein. Interestingly, the ureter was ligated and not reconnected to the bladder to reduce morbidity; moreover, it was also stated that no urine production was expected. Another interesting result, considering the whole organ for orthotopic transplantation and verifying its main function of urine production, was obtained by harvesting rat kidneys and preserving the intact and perfusable vascular, glomerular, and tubular compartments [136]. Kidneys, decellularized by a perfusion approach, were* in vitro* repopulated with endothelial and epithelial cells with consequent formation of a functional graft. Urine production was observed* in vitro* and this led the authors to test* in vivo* the engineered organ after orthotopic transplantation. Kidneys were anastomosed to the recipient's renal artery and vein and no evidence of bleeding was observed; ureter remained cannulated for collection of urine production, verified shortly after the unclamping of recipient vasculature. In addition *β*-1 integrin expression in engrafted podocytes suggested site-specific cell adhesion to physiologic ECM domains. This report highlighted the role of native ECM proteins, as laminins and collagen IV, the major ECM proteins of the glomerular basement membrane necessary for podocyte adhesion, slit diaphragm formation, and glomerular barrier function.

## 10. Bladder

Native nonurologic tissues, like gastrointestinal segments, are usually employed in reconstructive surgery [139, 140]. However, these tissues are lined by an absorptive and mucus-secreting epithelium that is incompatible with long-term exposure to urine [141]. This consideration further supports the need of a specific engineered scaffold that could properly replace the function of the organ, critically considering the microstructure of the native bladder which is then related to its specific action. Reported findings suggest that urothelium presents mechanosensitive sodium ion channels as transducers of the parasympathetic nervous system involved in bladder sensation and that cyclical deformation induces connective tissue synthesis [142]. The urothelium is one of the three major layers that constitute the bladder wall along with the lamina propria and the detrusor muscle. The different structures suggest that each component of this composite organ plays a peculiar role to assure the final function. In fact, an* ex vivo* study on the deformation characteristics of rat bladder wall showed that the organ can accommodate very large stretches prior to activation of neurological signals that trigger voiding and before collagen coils are fully distended, confirming that the lamina propria is the major structural capacitance layer, while the detrusor limits the total volume to avoid overdistention [143].

### 10.1. Preclinical Studies

#### 10.1.1. Natural Approach

SIS has been implanted in rat and canine bladder augmentation models, resulting in the regeneration of full thickness bladder tissue, including urothelium, muscularis, nerves, and blood vessels, characterized by physical properties, such as contractility, similar to native bladder [144]. Moreover, it has been demonstrated that terminally differentiated urothelium is the first portion of the bladder to completely regenerate after SIS implantation [145]. However, SIS, derived from different segments (proximal small bowel or distal ileum), resulted to have different regenerative potential and further investigation resulted to be necessary to identify the effective SIS regenerative potential [146].

Rabbit or sheep decellularized gallbladders (2 cm × 2 cm), either alone or seeded with autologous detrusor muscle small fragments, have been implanted on rabbit bladder mucosa for bladder augmentation. No evidence of inflammatory response was revealed 12 weeks postoperatively, and after 24 weeks seeded grafts resulted in whole bladder and muscular layer regeneration and in new micro vessel formation, suggesting that fragment-seeded cholecyst-derived ECM can enhance the properties of acellular gallbladder in terms of bladder wall regeneration [147]. Even if a decellularized scaffold seems to be the direct and straightforward solution for tissue engineering of tissues and organs, several issues need to be addressed as well. Probably, the most critical one is related to neovascularization, which is pivotal in promoting tissue formation and should be therefore favoured by developing ad hoc strategies. Kanematsu et al. [148] dealt with a rat bladder acellular matrix, as a model, incorporating bFGF by a reswelling procedure of the growth factor solution. When tested for a possible bladder augmentation in rats (diameter 15–20 mm), it was observed that bFGF significantly enhanced angiogenesis, probably inhibiting the graft shrinkage at 4 weeks in a dose dependent manner, which was not suppressed after 12 weeks. The authors reported that this occurrence could be related to regression of immature vessels formed by bFGF or unclear characteristics of the infiltrating cells. The strategy to include relevant chemical cues into the tested matrix was also verified by using VEGF, a well-known angiogenic factor. In this regard, porcine bladder was firstly decellularized and treated either with HA or with HA-VEGF and then implanted into pigs for bladder augmentation (4 × 4 cm^2^) [149]. The incorporation of HA significantly decreased the matrix porosity, preventing urine seepage and possible inflammation, while the presence of VEGF contributed to a great infiltration of microvessels. Moreover, the highest epithelialization was detected for the HA-VEGF scaffolds, as demonstrated by the presence of UPIII, a urothelium-specific protein. Subsequently, Chen et al. [150] proposed an acellular porcine bladder matrix preseeded with modified endothelial progenitor cells (transfected with VEGF gene carried by adenovirus). The scaffolds were then implanted into pigs which underwent partial cystectomy (about 40%); unseeded scaffolds were implanted as control. Postoperatively, treated grafts showed an enhanced neovascularization and prevented tissue shrinkage and scar formation.

Another possible concern on the use of naturally derived scaffolds is generally related to their limited temporal stability, but it has been rarely assessed* in vivo*. In this regard, ^14^C labelled piglet SIS sheets were implanted into dogs to replace between 35 and 45% of the dome of native bladders [151]. Liquid scintillation assays showed that less than 10% of the radioactivity remained at 3 months after surgery, in addition the excretion of the scaffold was mainly via a hematogenous route with subsequent urinary excretion.

#### 10.1.2. Synthetic Approach

According to the whole tissue engineering paradigm, an engineered scaffold can be cell seeded and conditioned, dynamically or not, before implantation. This option is not always followed as the implantation of the nude scaffold can be an alternative route to overcome the* in vitro* period that might expose the device to a possible contamination, also allowing reducing the waiting time before surgery. In this regard, the potential of noncell seeded gel spun silk matrices was assessed for murine bladder augmentation [152]. Silk fibroin solutions were prepared from* Bombyx mori* silkworm cocoons to obtain tubes by using the gel spinning technique. Tubular scaffolds were bisected along the central axis before implantation into an immune competent mouse strain model. Bladder reconstruction with silk led to an 82% survival rate, comparable to that with PGA (71%), and substantially higher than SIS implants (66%), the latter two being the reference cases. Connective tissue ingrowth was observed along the periphery of the silk matrix and traversed the defect site (day 21); the regenerated tissue was lined with a luminal multilayer epithelium bordered by a lamina propria with fibroblastic cell populations and a gradual increase in the degree and distribution of uroplakin expression was detected up to 70 days following implantation. However, a microscopic analysis of the implanted tube was not reported in the cited paper, as it was presented in a previous one [153]. As stated by the authors, no modifications occurred in the fabrication process and the final scaffold appeared to be like a dense structure without a fibrous architecture similar to the bladder ECM. The positive result presented seemed not to be affected by this occurrence, and probably an* in vivo* experimentation in larger animal models could further elucidate the point. Seeded polymeric scaffolds were evaluated in beagle dogs, undergone trigone-sparing cystectomy, by implanting PGA matrices (15 *μ*m average fiber diameter) coated with PLGA [154]. Urothelial and muscle cells were harvested, expanded separately, and, within 5 weeks, seeded onto biodegradable polymer matrices. Three different groups were considered for the study: (i) animals that were primarily closed gained a minimal amount of reservoir volume without regaining the precystectomy values, (ii) unseeded grafts led to a slight increase in volume, a well-developed urothelial layer and a deficient muscular architecture, (iii) seeded scaffolds allowed to approach and surpass the precystectomy bladder capacities, showing a normal cellular organization. Bladder cystoplasty was performed into mongrel dogs by implanting PLGA based scaffolds with or without autologous UCs and SMCs to assess the resulting outcome as affected by the presence of seeded cells [155]. In this regard, a regenerative process was observed when UCs and SMCs were seeded onto the implant, while a reparative healing occurred for the unseeded scaffolds (e.g., mucosal growth, but an incomplete tissue layer development). However, this study was not focused on the characteristics of the implanted scaffolds, thus not allowing evaluating the possible influence of the three-dimensional architecture on the final result. The influence of seeded or unseeded PGA scaffolds on urothelial proliferation was also previously assessed by using 3T3 mouse fibroblasts for rat bladder augmentation [156]. Seeded cells acted as a “feeder layer” confirming an improved outcome compared to the control case. Different cell types were also evaluated for this aim, as tested by Lai et al. [157] comparing bladder SMCs and intestinal SMCs seeded onto unwoven PGA sheets covered with PLGA for urinary bladder wall replacement in a rabbit model. However, a real improvement in bladder regeneration is strictly related to the nervous tissue regeneration. This issue was addressed by implanting PGA grafts, covered with fibroblast-seeded chitosan and modified with chitosan, to allow nerve growth within the bladder wall [158]. No complications were noticed and immunostaining results showed the presence of neuronal cells, suggesting that chitosan could improve scaffold abilities not only as a cell matrix, but also in guiding neurons into the graft.

In order to be as close as possible to the morphological structure of the bladder ECM, the fibrous architecture was considered by Del Gaudio et al. [159] evaluating an electrospun scaffold made of PCL and PHBV (50 : 50 ratio). The collected mats were composed of randomly arranged fibers, free of defects (3.0 ± 0.1 *μ*m average fiber diameter), and, due to the dynamic function of the here considered organ, characterized by means of dynamic mechanical analysis in a frequency range representative of the time scales of the physiological dynamics of the bladder wall. The electrospun scaffolds were implanted into rats for bladder augmentation and retrieved at 15, 30, and 90 days for the subsequent histological assays. The regenerative process was poorly evident in specimens 15 days after surgery, evident after 30 days and complete three months after. A progressive reconstitution of the entire bladder wall on the top of the scaffold to build a “new dome” was observed, with moderate scars along the suture lines. The bladder wall was reconstituted with the growth of both normal urothelium and fibrils of smooth muscle cells. The electrospinning approach was also followed to fabricate a hybrid scaffold composed of pig BAM substrates covered by PGA or PGA-PEG fibers [160]. An* in vivo* evaluation was carried out by implanting bladder SMC-seeded scaffolds into rats subjected to partial cystectomy (>50%): both types of substitutes supported the formation of a bladder wall-like structure, even if a significant shrinkage was observed for the PGA scaffold and an increased microvessel density characterized the PGA-PEG scaffold. Previously, the same group started this study including PLGA only and evaluating three different electrospinning procedures, that is, continuous spinning of PLGA microfibers on dry BAM, continuous spinning of PLGA microfibers on wet BAM, and layer-by-layer spinning of PLGA fibers on continuously rehydrated BAM [161]. After* in vivo* implantation, urothelium, smooth muscle, and collagen rich layers infiltrated with host cells and microvessels were detected. Furthermore, hybrid scaffolds maintained normal bladder capacity, whereas BAM recipients showed a significant distension of the bladder, demonstrating that adaptable hybrid scaffold supports bladder regeneration and holds potential for engineering of bladder.

A dome-shaped scaffold composed of electropulled PLGA mat (inner surface) and PLGA sponge, made by solvent casting and salt leaching, (outer surface) was evaluated as a potential substrate for amniotic stem cell differentiation into SMCs for rat bladder augmentation [162]. The composite had a final diameter of 10 mm and thickness of 0.8 mm; the inner surface was composed of polymeric fibers characterized by a large distribution (0.5–10 *μ*m). Postoperatively, bladder capacity and compliance were maintained in the cell-seeded group throughout the 12 weeks, while the acellular scaffold group (control group) showed a sequential deterioration with time. In addition, the contractile response was observed after 12 weeks, probably due, according to the authors, to the time for PLGA to be completely resorbed, as well as for the time required for the smooth muscle to gain significant mass.

Finally, to improve the* in vivo* outcome of the regenerative strategy, postfabrication treatments or scaffolds with nanostructured surface have been tested. Modified silk scaffolds have been implanted in a murine model for bladder augmentation and different results have been obtained, demonstrating that selective alterations in fabrication parameters can enhance the degradation rate of gel spun silk scaffolds* in vivo* while preserving their ability to support bladder tissue regeneration and function [163]. Nanometric (less than 100 nm) PLGA/PU scaffolds, treated with NaOH or HNO_3_ [164] and/or functionalized with IKVAV and YIGSR peptides, to improve cellular responses [165], have been tested for bladder tissue regeneration in a minipig model. After 11 weeks, both the urothelium and smooth muscle were consistently and continuously formed on the new bladder wall tissue with the polymer incorporation. These studies suggested that nanostructured resorbable synthetic scaffolds could be promising replacement materials for partial bladder and urogenital repair as well as neobladder replacement.

### 10.2. Clinical Studies

#### 10.2.1. Synthetic Approach

Moving to clinical applications, a viable engineered substitute should be fabricated based on the composite nature and properties of the bladder. This approach was followed by Atala et al. [17] to treat seven patients with myelomeningocele. Urothelial and muscle cells, obtained by a biopsy from each patient, were cultured and then seeded onto biodegradable bladder-shaped scaffold made of collagen (from homologous decellularized bladder submucosa), or collagen and PGA, to be implanted with or without omental wrap. In the follow-up no complications were noted and the renal function was preserved. Moreover, the mean maximum capacity in the collagen engineered bladders without omental wrap showed a 30% decrease, the one with omental wrap showed a 1.22-fold increase in volume, while the composite engineered bladders wrapped with omentum showed a 1.58-fold increase. These results supported the conclusion that PGA contributed to structural integrity, while collagen to cell growth and survival.

## 11. Urinary Tracts

In this section tissue engineering applications to both ureter and urethra reconstruction are considered. These structures are mainly characterized by a transport function that can be severely impaired by several disorders such hypospadias, epispadias, and strictures, specifically referring to urethra [166]. In this regard, the implanted scaffold should promptly concur to recover the natural function offering a suitable environment that does not elicit any adverse reaction, particularly due to the toxic nature of the fluids in contact with the scaffold surface. The role of a functional epithelium is therefore crucial and this might suggest planning ad hoc strategy for the development of a proper tissue engineered conduit.

### 11.1. Preclinical Studies

#### 11.1.1. Natural Approach

A possible improvement for urethral reconstruction, firstly based on the use of oral keratinocytes seeded onto BAM [167], was proposed by the same group considering oral keratinocyte and TGF-*β*1 siRNA transfected fibroblast seeded onto BAM in order to minimize the inflammatory response and avoid graft contraction and shrinkage [168]. For this aim, a mucosal defect (2 cm length and 0.8 cm width) was created in the anterior rabbit urethra obtaining 3 groups: oral keratinocyte and TGF-*β*1 siRNA transfected fibroblast-seeded BAM, autologous oral keratinocyte-seeded grafts, and unseeded grafts. All the animals survived to the postoperative observational period (up to 6 months), being characterized by a severe fibrosis and shrinkage (control group), intact epidermal cellular layer (increasing to 5–7 layers) with no evidence of the formation of capillary in the lower layer (autologous oral keratinocyte-seeded grafts), or by a well-developed 5 to 7 layers of stratified keratinocytes associated with the formation of capillary in the epithelial lower layer at 6 months after implantation (TGF-*β*1 siRNA group). This approach showed that TGF-*β*1 siRNA can inhibit the expression of TGF-*β*1 and significantly reduce the secretion of type I collagen and scar formation. The concept to use two different cell lines was already explored by Feng et al. [169], stating that this approach can accelerate the regenerative process and avoid strictures. In this regard, porcine ACSMs were seeded with lingual keratinocytes or with autologous CSMCs and lingual keratinocytes and transplanted into rabbits to recover a urethral defect. After 6 months, fibrosis, inflammation, and the absence of epithelium were observed in animals treated with ACSMs, simple epithelial layer regeneration was revealed in the case of ACSMs seeded with CSMCs, while stratified epithelial layer and organized muscle fiber bundles were evident in animal treated with ACSMs seeded with both cellular types. Even if a naturally derived material seems to be the most appropriate choice for a tissue engineered specific scaffold, several drawbacks can be highlighted as well. In fact, in this study a sever inflammatory response was highlighted due to (i) a slight retention of cellular compounds within the ACSM, that might cause chronic immunoreactions and fibrosis, (ii) the long urethral defect length (1.5 cm) that limited the regeneration of native urothelium, and (iii) the thickness of the scaffold that hindered the penetration of a vascular network into the matrix. A nude substrate can be hardly considered as a suitable means to promote tissue regeneration, since the lack of a functional epithelium concurs to the high rate urethral reconstruction failure. For this aim, Li et al. [170] assessed whether Epith-rASCs seeded onto rabbit bladder acellular matrix could aid the regeneration of a defect (2 cm length and 0.8 cm width) in rabbits' ventral anterior urethra. A remarkable stricture was detected into two control groups (undifferentiated adipose-derived stem cells grafts and unseeded grafts), differently from the treated case. Epith-rASCs showed the capability to differentiate into epithelium due to the contribution of* in vivo* urethral microenvironment. On the other hand, in the undifferentiated group, the contracture of grafts did not ameliorate even if epithelial differentiation of the implanted cells occurred, but this relatively slow process could not prevent inflammatory cell infiltration and fibrosis of lumen. The role of the epithelium was underlined by a comparable approach for the urethral reconstruction by using bone marrow MSCs and SMCs into a bladder acellular matrix [171]. Bladder was firstly explanted from rabbits and, after being decellularized, seeded with those two cell lines, wrapped around a catheter (MSCs on the luminal side), and covered with omentum has been implanted into rabbits to recover a 4 cm ureter defect. Multilayered urothelium covered the entire lumen with central visible neovascularization after 8 and 16 weeks postoperatively. This result further underlined the role of a proper scaffold, as the bladder acellular matrix, which provides at least 10 different biological factors (including, e.g., VEGF, TGF-*β*1, and bFGF), supporting a good microenvironment for cell migration, proliferation, and differentiation [172].

#### 11.1.2. Synthetic Approach

An example of urethral reconstruction by using a collagen tubular scaffold was proposed by Micol et al. [166]. According to this approach a high density collagen gel tube was fabricated including autologous SMCs from bladder biopsy to repair a 1 cm defect in the rabbit urethra. Spontaneous urothelial regeneration was observed that can be ascribed to colonization from the edges of native urothelium adjacent to the graft, or by seeding, during voiding, of urine-derived progenitor cells.

### 11.2. Clinical Studies

#### 11.2.1. Synthetic Approach

Clinical urethral reconstruction was performed in five boys (median age 11 years) by using tubularised fibrous polyglycolic acid:poly(lactide-coglycolide acid) scaffolds seeded with muscle (outer surface) and epithelial (inner surface) cells from a biopsy of each patient [20]. Scanning electron microscopy revealed that all scaffold surfaces were covered with cells at day 6 of culture. The size of the engineered urethras ranged from 4 to 6 cm (median 5 cm), with a 16 French diameter, and the construction of the implantable scaffolds took 4–7 weeks. All patients were continent and the absence of fistulae or urinary tract infections was verified during the follow-up (range 36–76 months).

## 12. Skeletal Muscle

Skeletal muscle has a remarkable inherent ability to regenerate and self-repair in response to limited and not severe injury and stress [173, 174]. Damage caused by crush injury or blunt trauma is typically associated with a host response that leads to the activation and differentiation of a population of resident muscle stem cells named satellite cells [175]. Therapeutic options for severe and extensive muscle injury (such as VML) are currently limited and reconstruction of skeletal muscle in these injuries remains a complex and unsolved task. Typical therapeutic procedures include autologous tissue transfer, muscle transposition, or amputation, but they show minimal success and extensive donor site morbidity [176–181]. Repair technologies have been focusing in the last few years on tissue engineering to reconstruct and restore structural and functional deficit of the skeletal muscle after VML. Several synthetic and natural-derived scaffolds have been developed and studied* in vitro* with the aim of mimicking the muscle ECM structure and properties; however, very few have been tested in reliable muscle injury animal models, mainly after total or partial muscle ablation and substitution [182].

### 12.1. Preclinical Studies

#### 12.1.1. Natural Approach

Several ECM proteins have been adopted for scaffold fabrication for skeletal muscle tissue engineering. Collagen electrospun scaffolds, seeded with C2C12 cells, were implanted in the mouse gastrocnemius. After different time points, a slow degradation of the scaffold has been showed, with activation of vascularization and the regeneration of muscle fibers [183]. These constructs, however, had low biocompatibility since only immune-compromised mice were able to integrate the 3D tissue graft into their host tissue. Also, the development of a fibrotic tissue has been shown and this is known to lead to healing problems and potential tissue/organ dysfunction during the final healing stages.

Fibrin, collagen, and other ECM proteins are used also to form scaffolds in commercially available gel forms or can be suspended for the creation of functional engineered musculoskeletal tissue [184]. These ECM-derived gels provide natural-like microenvironment for MSC proliferation and differentiation, but scale-up of these scaffolds is a major limitation.

A fibrin-based scaffold, seeded with adult human myoblasts, has been studied* in vivo* in a partial-thickness tibialis anterior muscle injury model in mouse [185]. Cell engraftment was observed 10 weeks after implantation, with reduced collagen deposition and mean tetanic force of the tibialis anterior recovered to approximately 90% of uninjured values. In a quite innovative approach, dual layered collagenous scaffolds were constructed with a radial pore orientation or with round pores. Unseeded scaffolds were implanted into a surgically created diaphragm defect in rats and explanted after 12 weeks. While new collagen deposition was more oriented in radial scaffolds with respect to round pores, cells and vessel migration within the scaffolds was similar between the two constructs [186].

Skeletal muscle regeneration and reconstruction are improved when native-derived scaffolds are used because of their content in cytokines that are released by means of matrix degradation. Furthermore, macrophage activation and polarization to M2 phenotype have been identified as an important mechanism determining proregenerative response after ECM-derived scaffold transplantation [187–191]. Acellular tissue scaffolds for skeletal muscle tissue engineering have been developed with several protocols starting from a wide variety of tissues and evaluated in a large number of animal models. ECM-derived scaffolds have been transplanted with and without muscle precursor cells since the early studies. An abdominal wall defect model has been used to test muscle-derived matrices obtained with different decellularization protocols and transplanted to correct the defect in rats and rabbits [192–194]. Matrices were also seeded with myoblasts and showed support to fibroblast migration, deposition of newly formed collagen, and neovascularization. However, evidence of skeletal-muscle-cell ingrowth was very poor and muscular electrophysiologic activity was minimum, with more encouraging results with myoblasts-seeded patches. Fibrosis development seems to be the major limitation of this approach using muscle-derived matrices after decellularization. Although acellular muscle ECM conserve chemical and architectural features of the original tissue, few papers highlighted strong myogenesis activation and de novo formation of muscle fibers derived from local activated muscle precursor cells [195–197].

As recently shown, the use of scaffolds originated from skeletal muscle tissue is not exclusive for muscle regeneration and different matrices can have equal or stronger benefits. SIS-derived acellular scaffolds have been compared* in vivo* to muscle ECM-derived matrices in a rat abdominal wall injury model [198]. Composition in growth factor content, GAGs, and basement membrane structural proteins were different between the two matrices, but* in vivo* results showed similar effects on constructive remodelling outcome and myogenesis in the implanted area, suggesting that superior muscle regeneration is not universally dependent upon homologous tissue derived ECM scaffolds [198, 199]. SIS-derived scaffolds have been largely tested in muscle defects in rodents and dogs, generally showing formation of vascularized skeletal muscle with different degrees of functional restoring (when tested) [181, 200, 201].

Porcine bladder-derived acellular matrix has also been tested in a mouse VML model with similar outcomes: 2 months postinjury and implantation of the scaffold seeded with muscle progenitor cells demonstrated remodelling of the construct and formation of new desmin-positive myofibers with and without striations, blood vessels, and neurovascular bundles in the site of implantation, but neuromuscular junction function has not been demonstrated [202]. Engineered acellular bladder seeded with muscle precursor cells and transplanted in a VML defect in tibialis anterior muscle exhibited variable capacity to restore* in vivo* function of injured muscles 12 weeks after injury [203]. In this work, cell seeded matrices promoted muscle fiber regeneration within the initial defect area, indicating that engineered constructs, including a cellular component, can improve the* in vivo* functional capacity of the injured musculature generating functional skeletal muscle fibers.

#### 12.1.2. Synthetic Approach

Several biodegradable synthetic scaffolds have been developed to support muscle regeneration and regrowth. Despite several attempts, cell engraftment, and proliferation within the synthetic construct is generally limited by low early adhesion. These scaffolds also tend to be more rigid than a biological matrix, interfering with functional muscle force development and transmission [184, 204]. Nevertheless, synthetic scaffolds are largely studied in literature for muscle tissue engineering application [184, 185, 205, 206].

Among several types of polymers used as a construct for muscle replacement and support, electrospun synthetic polymer mats, made of uniformly aligned fibers, resembling skeletal muscle ECM, are the scaffolds most evaluated in* in vivo* studies. Electrospun fibrous matrices have been fabricated of different single or combinations of polymers and promote cell adhesion and proliferation and nutrient diffusion and provide favourable mechanical properties [184, 205, 206]. These sheets of fibres from polymers, and sometimes biological proteins (elastin and collagen), are cost-effective and can be customized for pore size and fiber diameter and length and density. These characteristics affect muscle cell adhesion, migration, functional differentiation, and nutrient and oxygen availability in the engineered muscle [184, 205–207].

*In vivo*, unseeded biodegradable fibers of PEUU deposited with electrosprayed serum-based culture medium have been used in a rat model for abdominal wall replacement [208]. This wet electrospinning resulted in scaffolds with softer mechanical properties and a distinct morphology with fiber tortuosity (looping) that might ease cell migration by more readily locally distended fibers. 8 weeks after implantation, PEUU substrate showed a healing result that developed toward approximating physiologic mechanical behaviour. Extensive cellular infiltrate with smooth muscle and endothelial positive cells was observed together with ECM (collagens, elastin) elaboration. The functional result, however, seemed to be an effect of scaffold replacement by a fibrotic scar tissue improving only passive mechanical properties rather than active functionality of any new muscle tissue.

Zhao et al. [209] used an electrospun PCL/collagen hybrid scaffold for diaphragmatic muscle reconstruction. The aligned fibrous scaffold was fabricated to mimic muscle organization and to guide muscle cell orientation. Furthermore, these scaffolds, combining both synthetic polymer and naturally derived material, effectively supported cell adhesion, proliferation, and differentiation. The hybrid scaffolds were implanted into a central left hemidiaphragmatic defect in rats, showing muscle ingrowth into the scaffold up to 6 months after implantation. The mechanical properties of the retrieved diaphragmatic scaffolds were similar to those of normal tissue: electrospun PCL/collagen hybrid scaffolds exhibited initial elastic behaviour followed by stiffening, a behaviour which is similar to the tensile behaviour of native tissues. The* in vivo* remodelling process evidenced organized skeletal muscle cells, vascularization, and deep cell infiltration into the scaffold. Coaxial electrospun scaffolds, made of PCL, multiwalled carbon nanotubes and a (83/17 or 40/60) poly(acrylic acid)/poly(vinyl alcohol) hydrogel, capable of movement with electrical stimulation, have been implanted into a cavity (5 mm × 5 mm) created within the vastus lateralis muscle of rats for four weeks to determine* in vivo* biocompatibility. No significant clinical adverse effects and complications were revealed, and both scaffold types displayed evidence of muscle regeneration and neovascularisation after 28 days postimplantation. Although initial results are encouraging, authors commented that longer studies are necessary to determine if the two hydrogel concentrations will critically affect the body including local and systemic effects [210].

## 13. Concluding Remarks

The need for a suitable scaffold that can (i) accommodate cells, (ii) be instructive, (iii) mimic the natural ECM to be replaced, and (iv) promote an effective tissue regeneration is mandatory for tissue engineering applications. As already stated, this is only a partial vision of the whole problem because the term/concept “scaffold” needs to be understood in detail. The analysis of the results here reported, that was strictly focused on orthotopic implants with the aim to provide the state of the art for a reliable bench-to-bedside translation, clearly shows that multiple solutions have been proposed that still need to be properly refined for the desired outcome. Obviously, the* in vivo* approach does not guarantee a safe and viable technique to treat degenerative pathologies but is a necessary step to furnish relevant findings on the possible response of a tissue engineered scaffold. This approach helps to overcome the inherent limitations of the* in vitro* model but should not be considered as the only resource to test or verify an idea. On the contrary, it must be clearly stated that the animal experimentation is the last step to functionally assess a device when all the preliminary characterizations have given a positive response. As here demonstrated, dealing with scaffolds structurally similar to the anatomic site to be recovered, like the ones derived from a decellularization process, does not necessarily improve the host response since the key factor is the delicate interplay among different factors that concur to define a specific tissue. Morphology, three-dimensional architecture, mechanical and chemical properties, selection of the most appropriate cell type (i.e., stem cells or already differentiated cells), surface treatments, combination of multiple materials to fabricate a composite, loading and release of drugs and/or growth factors, and* in vitro*/*in vivo* models are to be critically evaluated and require a deep knowledge of all the aspects of the tissue or organ to be healed. The complexity of the problem is strictly dependent on the resulting outcome obtained by combining two or more of these starting elements, and a proper observational period after implantation can support the critical assessment of the investigated approach. Long follow-ups are therefore needed, like the one, for instance, ranging from 22 to 61 months considered to evaluate the response of tissue engineered bladders for patients needing cystoplasty [17] or the one (5 years) regarding the tissue engineered trachea transplantation to replace an end-staged left main bronchus with malacia [50].

Regarding the starting questions of this review, it is possible to conclude that clinical investigations effectively support the bench-to-bedside translation of the tissue engineering approach for some tissues/organs, allowing enhancing and proposing novel strategies toward a desired outcome. However, for complex organs more detailed studies are still necessary to develop a suitable engineered solution. A critical improvement of the knowledge of the host response to an implanted scaffold, which is dependent on all the features discussed in this review, is essential in order to accurately design a viable device aimed to readily promote the healing process.

## Figures and Tables

**Table 1:** Function and macroscopic structure of tissues and organs here considered, along with histological features and expected characteristics that an ideal tissue engineered substitute should provide for regeneration.

Organ	Function	Structure	Histological features	Ideal scaffold properties
Trachea	Conduction of air from the nose or mouth to the lungs	Thin-walled, fibromuscular, airtight tube supported by C-shaped, cartilaginous rings, spans by the pars membranacea (fibroelastic ligament). At its distal end, it bifurcates into the two main stem bronchi.	(i) Cartilaginous structure prevents collapse during respiration, provides flexibility, assures lumen patency; (ii) muscular tissue reduces lumen size during the cough reflex, facilitates airway clearance; (iii) mucosal membrane allows air conditioning, prevents epithelium dehydration	(i) lateral rigidity (ii) longitudinal flexibility (iii) impermeability to liquid and air (iv) ability to induce functional ciliated respiratory epithelium resurfacing (v) ability to induce blood vessel formation

Larynx	Orchestrate swallowing, breathing, coughing, and voice Immunological organ	Tuned sphincter situated in the anterior portion of the neck	Mucosa-covered collection of cartilaginous framework (three single and two paired cartilages), ligaments, muscles, and vocal cords (covered by respiratory epithelium)	(i) whole laryngeal framework (ii) low immunogenicity

Esophagus	Secretion of mucus to aid ingesta passage from the larynx to the stomach Move of ingesta to the stomach through peristaltic movement	Muscular tube	(i) Mucosa: basal membrane consisting of nonkeratinized squamous epithelial cells, which produce the mucus. (ii) Submucosa loose connective tissue (collagen types I and III, arranged in a criss-cross pattern), consisting of blood vessels and mucus glands. (iii) Muscularis externa: contains inner circular and outer longitudinal muscle cells (skeletal and smooth). (iv) Adventitia: composed of loose soft connective tissue, blood and lymph vessels, adipose tissue, and simple squamous cell epithelium	(i) tubular morphology and specific nanogeometry (four layers with different properties) (ii) resistant the reflux of gastric juice from the stomach (iii) appropriate mechanical properties (strength and viscoelasticity) to withstand peristaltic movements

Heart valves	Guarantee the unidirectional blood flow within the beating heart	Situated around a tendinous ring, possess three cusps, except for the mitral valve, only two.	Distinct complex layers composed of interstitial fibroblasts and connective tissue fibres and lined by valvular endothelial cells: (i) ventricularis composed of mainly aligned elastic fibers (assist valve leaflet dynamics) (ii) spongiosa consists mainly of proteoglycans and glycosaminoglycans (to absorb shear stress during cyclical valve motion) (iii) fibrosa composed of mainly collagen (provide strength and stiffness to maintain coaptation in the diastolic phase)	(i) highly specialized three-dimensional (inhomogeneous) microstructure (ii) anisotropic mechanical properties (iii) dynamic behavior

Vascular system (blood vessels)	Blood transport through the body	Fibromuscular tubular structure	Three layers (from the lumen outward): (i) tunica intima composed of an endothelial cell monolayer (diffusion of oxygen and carbon dioxide) (ii) tunica media composed of smooth muscle cells (regulate blood flow by altering vascular resistance through vasoconstriction and vasodilatation) (iii) tunica adventitia composed of fibroblasts and elastic connective tissue (stretches and supports blood vessels)	(i) longitudinal and transversal elasticity (ii) patency (iii) impermeability to liquid and air (iv) resistant to bacterial colonization (v) thrombotic formation resistance

Kidney	Maintain body homeostasis by excreting excess water, regulating the chemical blood composition, removing waste products, and assuring endocrinologic functions	Bean-shaped structure made of approximately 0.5–1 million nephrons, consisting of a glomerulus, surrounded by a Bowman's capsule, a proximal tubule, a loop of Henle, and a distal tubule connected to a collecting duct	Composed of various different cell types, including parietal cells, podocytes, tubule brush border cells, capillary bed covered by visceral epithelial cells, endothelial cells, and basement membrane (collagen IV, laminin, and heparin sulfate proteoglycans)	(i) provide blood ultrafiltration (ii) provide transport regulatory function (iii) appropriate mechanical properties (strength and viscoelasticity) to withstand peristaltic movements

Bladder	Store urine at low pressure and allow voluntary micturition, acting as a pressure vessel subjected to mechanical stress	Musculomembranous sac	(i) adventitia: connective tissue (ii) muscular layer: inner longitudinal, circular, and outer longitudinal layers of thick muscle bundles with intrafascicular connective tissue (iii) submucosa or lamina propria: connective tissue (collagen I, III, elastic fibers) (iv) mucosa: transitional epithelium (polyhedral flattened or large club-shaped cells), urothelial cells, and connective tissue (collagen IV and laminin) (v) serosa: simple squamous epithelium overlying connective tissue	(i) allow for even and constant attachment of mature epithelial cell layer on the luminal surface and harbor multiple cell layers of smooth muscle cells on the outside (ii) provide adequate mechanical support (iii) prevent collapse prematurely before new tissue *in vivo *formation
Urinary tracts	Propel urine from the kidneys to the bladder (ureters) and from the bladder to the outside of the body (urethra)	Narrow fibromuscular tubular structure		

Skeletal muscle	Locomotion, maintenance of posture, respiration (diaphragm and intercostal), communication, and production of body heat.	Composed of muscle cells (fibers), connective tissue, blood vessels, and nerves.	Muscle fibers form a long multinucleated syncytium grouped in bundles surrounded by connective tissue sheaths and extending from the site of origin to their insertion. Connective tissue covering: (i) epimysium: dense connective tissue ensheathing the entire muscle (ii) perimysium: surrounding bundles of muscle fibers (iii) endomysium: layer of reticular fibers and ECM surrounding individual muscle fibers.	(i) appropriate mechanical properties, such as contraction, stiffness, force, and elasticity (ii) provide orientation fiber guiding (iii) provide adequate porosity

## Acronyms

ACSM: Acellular corpus spongiosum matrix
ASCs: Adipose stem cells
BAM: Bladder acellular matrix
bFGF: Basic fibroblast growth factor
BMCs: Bone marrow cells
CSMCs: Corporal smooth muscle cells
ECM: Extracellular matrix
EPCs: Epithelial precursor cells
Epith-rASCs: Epithelial-differentiated rabbit adipose-derived stem cells
G-CSF: Granulocyte colony-stimulating factor
GAGs: Glycosaminoglycans
HA: Hyaluronic acid
MSCs: Mesenchymal stem cells
PCL: Poly(*ε*-caprolactone)
PCU: Poly(carbonate-urea)urethane
PEG: Poly(ethylene glycol)
PEUU: Poly(ester urethane)urea
PGA: Polyglycolic acid
PHBHHx: Poly(3-hydroxybutyrate-co-3-hydroxyhexanoate)
PHBV: Poly(3-hydroxybutyrate-co-3-hydroxyvalerate)
PLA: Polylactic acid
PLGA: Poly(lactic-*co*-glycolic acid)
POSS: Polyhedral oligomeric silsesquioxane
PU: Poly(ether urethane)
ePTFE: Expanded polytetrafluoroethylene
SDS: Sodium dodecyl sulphate
SIS: Small intestine submucosa
SMCs: Smooth muscle cells
TGF-*β*1: Transforming growth factor-*β*1
UCs: Urothelial cells
VEGF: Vascular endothelial growth factor
VML: Volumetric muscle loss.
